# Risk factors and outcomes associated with systolic dysfunction following traumatic brain injury

**DOI:** 10.1097/MD.0000000000038891

**Published:** 2024-07-26

**Authors:** Jungen Li, Yuzhu Miao, Guoxing Zhang, Xiaowen Xu, Yanxia Guo, Bingyuan Zhou, Tingbo Jiang, Shiqi Lu

**Affiliations:** a Department of Emergency, the First Affiliated Hospital of Soochow University, Suzhou, China; b Department of Echocardiography, the First Affiliated Hospital of Soochow University, Suzhou, China; c Department of Physiology and Neuroscience, Medical College of Soochow University, Suzhou, China; d Department of Emergency, Suzhou Municipal Hospital of Nanjing Medical University, Suzhou, China; e Department of Critical Care Medicine, the First Affiliated Hospital of Soochow University, Suzhou, China.

**Keywords:** echocardiography, heart rate, hypersensitive cardiac troponin T, prognosis, systolic blood pressure, systolic dysfunction, traumatic brain injury

## Abstract

Systolic dysfunction has been observed following isolated moderate–severe traumatic brain injury (Ims-TBI). However, early risk factors for the development of systolic dysfunction after Ims-TBI and their impact on the prognosis of patients with Ims-TBI have not been thoroughly investigated. A prospective observational study among patients aged 16 to 65 years without cardiac comorbidities who sustained Ims-TBI (Glasgow Coma Scale [GCS] score ≤12) was conducted. Systolic dysfunction was defined as left ventricular ejection fraction <50% or apparent regional wall motion abnormality assessed by transthoracic echocardiography within 24 hours after admission. The primary endpoint was the incidence of systolic dysfunction after Ims-TBI. The secondary endpoint was survival on discharge. Clinical data and outcomes were assessed within 24 hours after admission or during hospitalization. About 23 of 123 patients (18.7%) developed systolic dysfunction after Ims-TBI. Higher admission heart rate (odds ratios [ORs]: 1.05, 95% confidence interval [CI]: 1.02–1.08; *P* = .002), lower admission GCS score (OR: 0.77, 95% CI: 0.61–0.96; *P* = .022), and higher admission serum high-sensitivity cardiac troponin T (Hs-cTnT) (OR: 1.14, 95% CI: 1.06–1.22; *P* < .001) were independently associated with systolic dysfunction among patients with Ims-TBI. A combination of heart rate, GCS score, and serum Hs-cTnT level on admission improved the predictive performance for systolic dysfunction (area under curve = 0.85). Duration of mechanical ventilation, intensive care unit length of stay, and in-hospital mortality of patients with systolic dysfunction was higher than that of patients with normal systolic function (*P *< .05). Lower GCS (OR: 0.66, 95% CI: 0.45–0.82; *P *= .001), lower admission oxygen saturation (OR: 0.82, 95% CI: 0.69–0.98; *P* = .025), and the development of systolic dysfunction (OR: 4.85, 95% CI: 1.36–17.22; *P* = .015) were independent risk factors for in-hospital mortality in patients with Ims-TBI. Heart rate, GCS, and serum Hs-cTnT level on admission were independent early risk factors for systolic dysfunction in patients with Ims-TBI. The combination of these 3 parameters can better predict the occurrence of systolic dysfunction.

## 1. Introduction

Traumatic brain injury (TBI) is the leading cause of mortality in young adults and a major cause of death and disability across all age groups,^[1]^ particularly in middle-income countries.^[2]^ At present, treatment of TBI presents many problems and challenges, especially in cases of moderate and severe TBI. The most important aspects of TBI treatment are controlling intracranial pressure (ICP) and maintaining appropriate cerebral perfusion pressure.^[3]^ TBIs may disrupt cardiac output, and the resultant alterations in cerebrovascular circulation can result in secondary brain injury.^[4]^ Several studies have demonstrated that patients with TBI and other neurological injuries, such as subarachnoid hemorrhage, may develop hypertension and systolic dysfunction, and this is associated with increased mortality or other poor outcomes.^[5]^ Although some studies have focused on myocardial injury associated with TBI, the potential factors affecting systolic dysfunction after TBI and the influence of systolic dysfunction on the prognosis of patients with TBI are still unclear. Therefore, we conducted a prospective observational study to improve our understanding of the mechanisms underlying systolic dysfunction following TBI in order to provide potential therapeutic targets for preventing the occurrence of systolic dysfunction and improving prognosis after TBI.

## 2. Methods

### 2.1. Study design and participants

This was a single-center prospective observational study performed at the First Affiliated Hospital of Soochow University, a tertiary hospital in China, and was approved by the Ethics Committee of the First Affiliated Hospital of Soochow University. All patients, or the patient’s next of kin, were informed about the study and provided informed consent.

In the present study, we included all consecutive adult patients aged ≥16 years with a moderate–severe (postresuscitation Glasgow Coma Scale [GCS] of 3–12) TBI who were admitted to the trauma center of our hospital from January 2018 to December 2019. According to the literature,^[6]^ we restricted our study population to isolated moderate–severe traumatic brain injury (Ims-TBI), defined as nonhead Abbreviated Injury Scale score <3 in other body regions. Furthermore, we excluded patients who were older than 65 years, patients who had a history of cardiac arrest, known ischemic or structural cardiac disease, severe medical comorbidities (liver cirrhosis, chronic kidney disease, human immunodeficiency virus, history of chemotherapy, chronic obstructive pulmonary disease, pulmonary hypertension, and history of cerebrovascular disease), and patients requiring more than 2 units of packed red blood cells as part of their initial resuscitation in the emergency department and/or operating room.

### 2.2. Assessment of cardiac systolic dysfunction

Transthoracic echocardiography was performed by a cardiac sonographer at the bedside using a Vivid IQ cardiovascular color ultrasound (GE) and 2-dimensional ultrasound and Doppler technology according to the guidelines of the American Society of Echocardiography within the first 24 hours after admission.^[7]^ According to the literature,^[8]^ systolic dysfunction is defined as left ventricular ejection fraction (LVEF) (using 2-and 4-chamber view biplane Simpson method) <50% or apparent regional wall motion abnormalities (grade 0 = none, 1+ = hypokinesis, 2+ = severe hypokinesis, 3+ = akinesis, and 4+ = dyskinesis). The cardiac sonographer was not aware of the clinical conditions of the patients, and the echocardiographic data of all patients were collected for analysis. Insufficient image data owing to echocardiography not being able to assess systolic function were excluded.

### 2.3. Data collection and outcomes

Venous blood was drawn from the cubital vein within 6 hours of admission to assess high-sensitivity cardiac troponin T (Hs-cTnT) levels using a cobas e 411 ElectroChemiLuminescence immunoassay analyzer (Roche). The following patient data were collected: age, sex, admission GCS, admission systolic blood pressure, admission heart rate, injury mechanism, initial head computed tomography findings, admission oxygen saturation, admission hematocrit, and clinical treatment variables (intracranial procedures, analgesia, sedation, etc). Data concerning the primary outcome (incidence of systolic dysfunction after TBI within 24 hours of admission) and secondary outcomes (duration of mechanical ventilation, intensive care unit [ICU], hospital length of stay [LOS], and hospital mortality) were collected.

### 2.4. Clinical care

Resuscitation of patients is based on relevant TBI clinical treatment guidelines.^[9]^ Clinical care included ensuring an open airway, providing mechanical ventilation if necessary, maintaining an arterial partial pressure of carbon dioxide of 35 to 40 mm Hg and hemodynamic stability when it occurs, norepinephrine should be used to maintain systolic blood pressure >90 mm Hg during hypotension, controlling intracranial pressure (ICP < 25 mm Hg), maintaining proper cerebral perfusion pressure (50–70 mm Hg) and body temperature (35–37.5°C) (antipyretic drugs or physical cooling measures should be used if necessary), and initiating analgesia and sedation and osmotic therapy (mannitol or hypertonic saline) if necessary. If surgical indications were present, intracranial procedures including ICP monitor placement and/or intracranial surgery were performed. Other treatment measures included prevention and treatment of infections, nutritional support, and maintenance of internal environment stability of the body.

### 2.5. Statistical analysis

Continuous variables with symmetric distribution were presented as the mean ± standard deviation, while variables with asymmetric distribution were presented as median and interquartile range. Categorical variables were expressed as frequency and percentage. Continuous variables with a normal distribution were compared between groups using Student *t* test, and variables with a nonnormal distribution were compared using the Wilcoxon ranked test. Statistical significance of differences between categorical variables was evaluated using the chi-squared test. We included demographic and clinical characteristic variables with statistically significant differences (*P *< .05) between the systolic dysfunction group and the nonsystolic dysfunction group in the logistic regression analysis. Similarly, demographic and clinical characteristic variables with statistically significant differences (*P *< .05) between the survival group and the nonsurvival group during hospitalization were also included in the logistic regression analysis. Logistic regression was used to identify predictors for subsequent systolic dysfunction within 24 hours after TBI and for in-hospital mortality. The results were summarized by the corresponding odds ratio (OR) and adjusted OR with 95% confidence interval (CI). The efficiency of risk factors for predicting systolic dysfunction was evaluated by assessing the area under the curve (AUC) of the receiver operating characteristic curve. All analyses were conducted using IBM-SPSS version 25 (IBM corporation, Armonk), and *P *< .05 was considered statistically significant for all 2-tailed tests.

## 3. Results

### 3.1. Demographic data and clinical features

**Figure 1.**
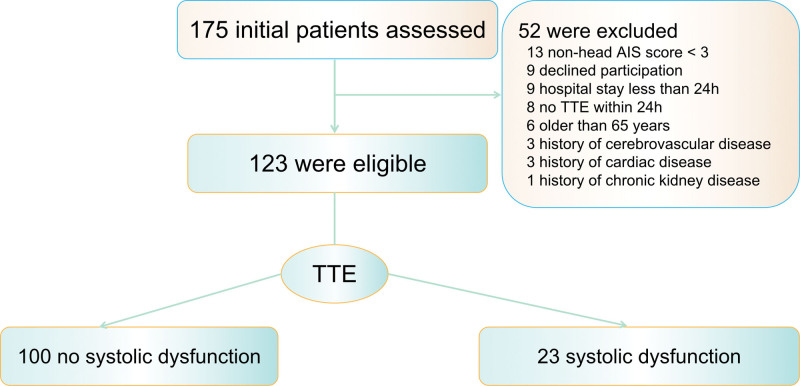
Flow of participants in the study. AIS, Abbreviated Injury Scale; TTE, transthoracic echocardiography.

In total, 175 patients met the inclusion criteria and 52 patients were excluded for the following reasons: refusal to participate in the study (n = 9), the presence of polytrauma with nonhead AIS score >3 (n = 13), hospital stay <24 hours (n = 9), older than 65 years (n = 6), presence of underlying cardiac disease (n = 3), presence of underlying cerebrovascular disease (n = 3), presence of underlying chronic kidney disease (n = 1), no transthoracic echocardiography within 24 hours following injury due to clinical circumstances (e.g., prolonged transport time, resuscitation, clinical procedures, or surgery) (n = 8). After these exclusions, 123 patients were considered eligible for analysis (Fig. 1). Of these, 23 (18.7%) patients developed systolic dysfunction and were classified into the systolic dysfunction group, while the remaining 100 patients were classified into the no systolic dysfunction group. The baseline demographic and clinical characteristics of all patients are shown in Table 1. The mean age of the patients was 41.1 ± 12.8 years, and most were men (77.2%). There were no differences between patients with and without systolic dysfunction in terms of age, presence of initial head computed tomography findings, and injury mechanism. Compared with patients who experienced normal systolic function, those with systolic dysfunction had significantly lower GCS (5 vs 8, *P* < .001) but had higher systolic blood pressure (145.4 ± 25.1 vs 134.9 ± 19.9 mm Hg, *P *< .05), higher heart rates (98.7 ± 27.3 vs 82.9 ± 12.0 beats/min, *P *< .001), higher use of sedative drugs (78.3% vs 54%, *P *< .05), and higher Hs-cTnT levels (17.4 ± 9.7 vs 8.9 ± 5.5 pg/mL, *P *< .001) at admission.

**Table 1:** Demographic and clinical characteristics of study participants.

Variable	All patients (N = 123)	No systolic dysfunction (n = 100)	Systolic dysfunction (n = 23)	*P*
Age (yr)	41.05 ± 12.8	40.58 ± 12.5	43.48 ± 13.9	.329
Male sex	95 (77.2%)	78 (78.0%)	17 (73.9%)	.673
Injury mechanism				
Traffic injury	78 (63.4%)	64 (64.0%)	14 (60.9%)	.779
Fall	29 (23.6%)	23 (23.0%)	6 (26.1%)	.753
Assault	9 (7.3%)	7 (7.0%)	2 (8.7%)	.675
Other	7 (5.7%)	6 (6.0%)	1 (4.3%)	.742
Initial head CT findings*				
Epidural hemorrhage	13 (10.6%)	10 (10.0%)	3 (13.0%)	.454
Subdural hemorrhage	102 (82.9%)	81 (81.0%)	21 (91.3%)	.359
Subarachnoid hemorrhage	100 (81.3%)	80 (80.0%)	20 (86.9%)	.556
Intraparenchymal hemorrhage	52 (42.3%)	40 (40.0%)	12 (52.2%)	.287
Admission GCS	8 (6–11)	8 (6–11)	5 (4–7)	**<.001**
Admission SBP (mm Hg)	136.9 ± 22.5	134.9 ± 19.9	145.4 ± 25.1	**.043**
Admission heart rate (beats/min)	86.0 ± 17.2	82.9 ± 12.0	98.7 ± 27.3	**<.001**
Admission oxygen saturation (%)	97.6 ± 3.2	97.2 ± 2.9	96.0 ± 4.5	.248
Admission Hct (%)	37.5 ± 4.0	37.4 ± 4.06	38.3 ± 3.6	.336
Admission Hs-cTnT (pg/mL)	10.7 ± 7.2	8.9 ± 5.5	17.4 ± 9.7	**<.001**
Vasopressor†	35 (28.5%)	27 (27.0%)	8 (34.7%)	.453
Intracranial procedures†				
ICP monitor placement	43 (35.0%)	34 (34.0%)	9 (39.1%)	.642
Need for intracranial surgery	44 (35.8%)	34 (34.0%)	10 (43.4%)	.393
Analgesia†‡	109 (88.6%)	86 (86.0%)	23 (100%)	.057
Sedation†§	72 (58.5%)	54 (54.0%)	18 (78.3%)	**.037**
Osmotherapy†∥	104 (84.6%)	82 (82.0%)	22 (95.6%)	.122
Fluid balance (mL)†				
Intake	3112.9 ± 504.9	3087.9 ± 515.7	3234.4 ± 436.8	.210
Output	3140.4 ± 621.5	3176.0 ± 729.7	2975.2 ± 436.7	.162

Values are mean ± standard deviation or mean (interquartile range) for continuous variables and n (%) for categorical variables.

bpm = beats per minute, CT = computed tomography, GCS = Glasgow Coma Scale, Hct = hematocrit, Hs-cTnT = high-sensitivity cardiac troponin T, SBP = systolic blood pressure.

* Some patients had multiple head CT findings.

† During the first 24 hours after admission.

‡ Any use of remifentanil or dezocine.

§ Any use of midazolam or propofol.

∥ Any use of hypertonic saline or mannitol.

### 3.2. Impact of systolic dysfunction after Ims-TBI on patient prognosis

Table 2 shows that compared with the no systolic dysfunction group, durations of mechanical ventilation (4.0 vs 0.5 days, *P *= .001), ICU LOS (12 vs 3 days, *P *= .001), and in-hospital mortality (52.1% vs 11.0%, *P *< .001) of patients with cardiac dysfunction after TBI were higher. Patients were divided into 2 groups according to survival; those who died during hospitalization were placed in the nonsurvival group and all others were placed in the survival group. Table 3 shows that patients in the nonsurvival group had lower GCS (4 vs 8, *P *< .001), higher incidence of cardiac dysfunction (52.1% vs 11.0%, *P *< .001), lower oxygen saturation (95.5 ± 4.2% vs 97.6 ± 2.7%, *P *= .010), and higher Hs-cTnT levels on admission (14.8 ± 9.1 vs 9.9 ± 6.4 pg/mL, *P *= .003). Multivariate regression analysis showed that low GCS score (OR: 0.66, 95% CI: 0.45–0.82; *P *= .001), low admission oxygen saturation (OR: 0.82, 95% CI: 0.69–0.98; *P *= .025), and systolic dysfunction (OR: 4.85, 95% CI: 1.36–17.22; *P *= .015) were independently associated with in-hospital mortality (Table 4).

**Table 2:** Outcomes of patients with and without systolic dysfunction after Ims-TBI.

Variable	All patients	No systolic dysfunction (n = 100)	Systolic dysfunction (n = 23)	*P*
Duration of mechanical ventilation (d)	2 (0–5)	0.5 (0–4)	4.0 (2.0–12.0)	**.001**
ICU LOS (d)	4 (0–13)	3 (0–12)	12 (4.0–19.0)	**.001**
Hospital LOS (d)	14 (9–21)	15 (10–20)	18.0 (9–33)	.209
In-hospital mortality, n (%)	23 (18.7%)	11 (11.0%)	12 (52.1%)	**<.001**

Values are mean (interquartile range) for continuous variables and n (%) for categorical variables.

ICU = intensive care unit, Ims-TBI = isolated moderate–severe traumatic brain injury, LOS = length of stay.

**Table 3:** Demographic and clinical characteristics of survivors and nonsurvivors.

Variable	Survivors (n = 100)	Nonsurvivors (n = 23)	*P*
Age (yr)	41.4 ± 13.0	40.5 ± 12.3	.739
Male gender	78 (78.0%)	17 (73.9%)	.784
Injury mechanism			
Traffic injury	64 (64.0%)	14 (60.8%)	.813
Fall	24 (24.0%)	5 (21.7%)	.818
Assault	7 (7.0%)	2 (8.6%)	.675
Other	5 (5.0%)	2 (10.8%)	.462
Initial head CT findings*			
Epidural hemorrhage	10 (10.0%)	3 (13.0%)	.708
Subdural hemorrhage	81 (81.0%)	21 (91.3%)	.149
Subarachnoid hemorrhage	82 (82.0%)	20 (86.9%)	.762
Intraparenchymal hemorrhage	40 (40.0%)	12 (52.1%)	.202
Admission GCS	8 (6–11)	4 (3–6)	<.001
Admission SBP (mm Hg)	135.6 ± 19.2	140.5 ± 29.5	.186
Admission heart rate	84.9 ± 16.3	90.4 ± 19.4	.132
Admission oxygen saturation (%)	97.6 ± 2.7	95.5 ± 4.2	.010
Vasopressor†	24 (24.0%)	11 (47.8%)	.024
Intracranial procedures†			
ICP monitor placement	33 (33.0%)	10 (43.5%)	.344
Need for intracranial surgery	33 (33.0%)	11 (47.8%)	.227
Analgesia†‡	88 (88.0%)	21 (91.3%)	.491
Sedation†§	55 (55.0%)	17 (73.9%)	.107
Osmotherapy†∥	83 (83.0%)	21 (91.3%)	.523
Fluid Balance (mL)†			
Intake	3090.8 (503.8)	3182.1 (504.3)	.755
Output	3115.7 (628.6)	3200.6 (598.2)	.503
Admission Hct (%)	37.6 ± 4.1	37.4 ± 3.8	.799
Admission Hs-cTnT (pg/mL)	9.9 ± 6.4	14.8 ± 9.1	.003
Systolic dysfunction n (%)	11 (11.0%)	12 (52.1%)	<.001

Values are mean ± standard deviation or mean (interquartile range) for continuous variables and n (%) for categorical variables.

bpm = beats per minute, CT = computed tomography, GCS = Glasgow Coma Scale, Hct = Hematocrit, Hs-cTnT = high-sensitivity cardiac troponin T, SBP = systolic blood pressure.

* Some patients had multiple head CT findings.

† During the first 24 hours after admission.

‡ Any use of remifentanil or dezocine.

§ Any use of midazolam or propofol.

∥ Any use of hypertonic saline or mannitol.

**Table 4:** Logistic regression analysis of risk factors for in-hospital mortality following TBI.

Variate	Univariate analysis			Multivariate analysis		
	OR	95% CI	*P*	OR	95% CI	*P*
GCS	0.64	0.47–0.87	**.005**	0.66	0.45–0.82	**.001**
Systolic dysfunction	4.46	1.13–17.5	**.032**	4.85	1.36–17.22	**.015**
Admission oxygen saturation	0.86	0.74–1.00	**.051**	0.819	0.69–0.98	**.025**
Hs-cTnT	1.02	0.94–1.10	.586			
Vasopressor	2.29	0.79–6.64	.125			

CI = confidence interval, GCS = Glasgow Coma Scale, Hs-cTnT = high-sensitivity cardiac troponin T, OR = odds ratio.

### 3.3. Independent risk factors for systolic dysfunction

The development of systolic dysfunction after Ims-TBI was set as the dependent variable and variables that were significant (*P *< .05) in univariate analysis were taken as independent variables. Upon logistic regression analysis, high heart rate on admission (OR: 1.05, 95% CI: 1.02–1.08; *P *= .002), low GCS score on admission (OR: 0.77, 95% CI: 0.61–0.96; *P *= .022), and high serum Hs-cTnT level on admission (OR: 1.14, 95% CI: 1.06–1.22; *P *< .001) were independently associated with the development of systolic dysfunction among patients with Ims-TBI (Table 5).

**Table 5:** Logistic regression analysis of risk factors for systolic dysfunction following TBI.

Variate	Univariate analysis			Multivariate analysis		
	OR	95% CI	*p*	OR	95% CI	*p*
GCS	0.77	0.61–0.98	**.033**	0.77	0.61–0.96	**.022**
Admission SBP	1.02	0.99–1.01	.171			
Admission heart rate	1.06	1.02–1.09	**.002**	1.05	1.02–1.08	**.002**
Hs-cTnT	1.13	1.05–1.21	**.002**	1.14	1.06–1.22	**<.001**
Sedation	1.40	0.36–5.43	.626			

GCS = Glasgow Coma Scale, Hs-cTnT = high-sensitivity cardiac troponin T, SBP = systolic blood pressure, TBI = traumatic brain injury.

### 3.4. Predictive value of admission-related parameters for cardiac systolic dysfunction in patients with Ims-TBI

**Figure 2.**
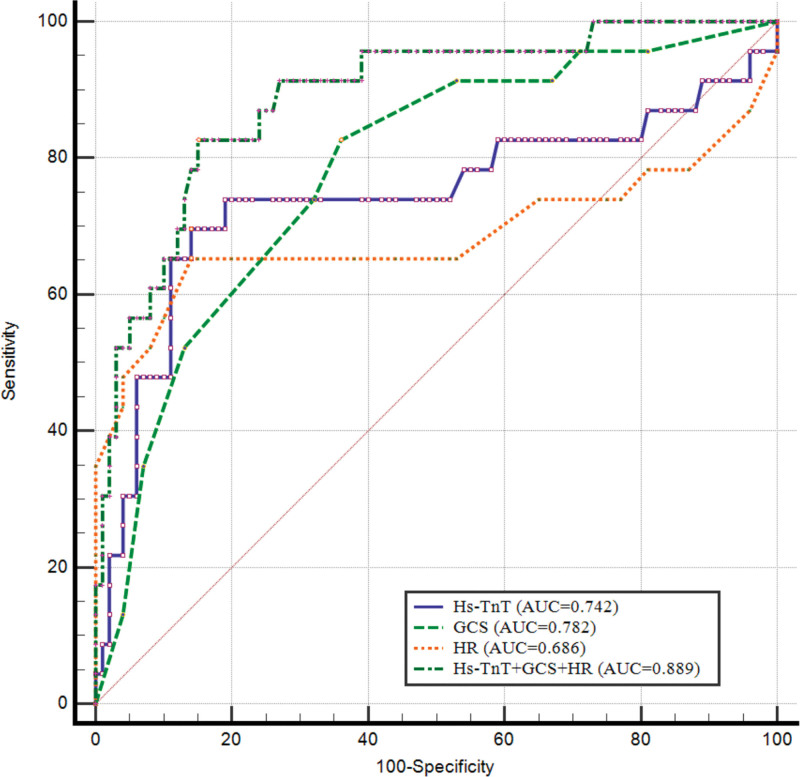
Receiver operating characteristic curve of clinical parameters. AUC, area under the curve; GCS, Glasgow Coma Scale; Hs-cTnT, high-sensitivity cardiac troponin T; HR, heart rate.

The AUC of admission serum Hs-cTnT level for predicting systolic dysfunction was 0.74 (95% CI: 0.66–0.82, *P* < .001) with an optimal cutoff of ≥13.3 mmol/L. The AUC of admission GCS score for predicting systolic dysfunction was 0.78 (95% CI: 0.70–0.85, *P *< .001) with an optimal cutoff of ≤7 points. The AUC of heart rate on admission for predicting systolic dysfunction was 0.69 (95% CI: 0.60–0.77, *P* = .030) with an optimal cutoff of ≥92 beats/min. A combination of GCS score, admission Hs-cTnT level, and heart rate improved the predictive performance for assessing systolic dysfunction (AUC: 0.85, 95% CI: 0.77–0.91, *P* < .001) (Table 6 and Fig. 2).

**Table 6:** Receiver operating characteristic curve of clinical parameters related to systolic dysfunction following Ims-TBI.

	AUC-ROC	95% CI	Cutoff	*P*	Se (%)	Sp (%)	+LR	−LR	−PV	+PV
Univariate										
Admission GCS	0.782	0.698–0.851	≤7	<.001	82.61	64.00	2.29	0.27	34.50	94.10
Hs-cTnT	0.742	0.656–0.817	≥13.32	<.001	69.57	86.00	4.97	0.35	92.50	53.30
HR	0.686	0.596–0.766	≥92	.030	65.22	86.00	4.66	0.40	91.50	51.70
Combined variate										
GCS + Hc-TnT + HR	0.848	0.772–0.906	≥-1.44	<.001	82.61	80.00	4.13	0.22	95.20	48.70

AUC = area under the curve, CI = confidence interval, GCS = Glasgow Coma Scale, HR = heart rate, Hs-cTnT = high-sensitivity cardiac troponin T, Ims-TBI = isolated moderate–severe traumatic brain injury, ROC = receiver operating characteristic, se = sensitivity, sp = specificity.

## 4. Discussion

In this study, we aimed to determine the risk factors for the development of systolic dysfunction after Ims-TBI and the impact of systolic dysfunction on the prognosis of patients with Ims-TBI. The primary findings of our study were that higher heart rate, lower GCS, and higher serum Hs-cTnT level on admission were independently associated with the development of systolic dysfunction in patients with Ims-TBI; a combination of heart rate, GCS, and serum Hs-cTnT level on admission improved the predictive performance for systolic dysfunction; higher in-hospital mortality and longer ICU LOS were found in the patients who developed systolic dysfunction after TBI; and the development of systolic dysfunction after TBI was an independent risk factor for in-hospital mortality. To our knowledge, this is the first prospective observational study performed in China to simultaneously investigate the risk factors of developing systolic dysfunction after Ims-TBI and the impact of systolic dysfunction on the prognosis of these patients.

TBI is commonly complicated by additional organ damage.^[10]^ Unfortunately, few studies have focused on TBI accompanied by systolic dysfunction. Systolic dysfunction after TBI is a form of stress cardiomyopathy^[11]^ and is common in patients with TBI. A cohort study showed that the incidence of systolic dysfunction after Ims-TBI was 22%.^[6]^ Another study found that 27% of patients with TBI developed systolic dysfunction during hospitalization, excluding those with preexisting heart disease.^[8]^ In our study, approximately 18.7% of patients with Ims-TBI who had no prior heart disease were found to develop systolic dysfunction defined by an LVEF <50% or apparent regional wall motion abnormality after injury.

At present, the understanding of the specific mechanism underlying early complicated systolic dysfunction in patients after TBI is still unclear. Previous studies have shown that cases of nontraumatic neurologic disease paradigms such as subarachnoid hemorrhage are often complicated by systolic dysfunction.^[12,13]^ The underlying mechanism may be due to increased intracranial pressure following neurologic injury leading to activation of neuroendocrine pathways, hyperexcitability of the sympathetic nerves, increased secretion of catecholamines, and cyclic adenosine monophosphate-mediated calcium ion overload. In addition, ischemic perfusion injury and necrosis of the contraction zone can in turn cause cardiac dysfunction.^[14–18]^ Krishnamoorthy et al found that the hemodynamic characteristics of patients with TBI complicated by systolic dysfunction included hypertension and tachycardia on admission and decreased blood pressure and heart rate 12 hours after admission.^[19]^ Similarly, our study findings also showed that the admission heart rate and systolic blood pressure in the systolic dysfunction group were higher than those in the no systolic dysfunction group. Several studies have shown that increased heart rate and blood pressure caused by the excessive release of catecholamines may be a precursor to cardiac systolic dysfunction and hemodynamic disturbances.^[5,17]^ The excessive release of catecholamines in patients with TBI within 48 hours is associated with a poor prognosis.^[20]^ In addition, a considerable number of studies have shown that using β-blockers to inhibit adrenergic activity in patients with TBI can mitigate the effect of excess catecholamine release, thereby improving the prognosis of patients with TBI.^[21]^ These results indicate that the adverse stress response in patients with TBI to early sympathetic excitement and excessive release of catecholamines may be the cause of the cardiac dysfunction in these patients. Excessive release of catecholamines usually leads to an increase in arterial blood pressure and heart rate, and high blood pressure can cause brain damage through vasogenic brain edema and damage to the myocardium.

GCS is the most commonly used index for evaluating the severity and prognosis of patients with TBI. At present, it is known that the central nervous system directly affects cardiac activity through the intrinsic conduction pathways (e.g., direct innervation) of the heart.^[13,22]^ The hypothalamus, substantia red nucleus system, and brainstem parasympathetic nucleus are all involved in regulating cardiac activity, and the pituitary-hypothalamus axis also plays an important role in regulating the function of the cardiovascular system.^[22]^ We observed that, compared with patients with no systolic dysfunction after Ims-TBI, patients with systolic dysfunction had more severe head injury (lower GCS). Further multivariate analysis showed that low GCS is an independent risk factor for patients with TBI and systolic dysfunction. This may be explained by activation of neuroendocrine pathways in the lower brain and hypothalamus after severe TBI, other brain injury, or intracranial hypertension. Activation of these pathways alters both, the direct and indirect regulation of the cardiovascular system by altering central nervous system function, leading to secondary myocardial damage.^[15]^

Troponin is a specific marker of myocardial injury. Previous studies have found that patients with acute brain injury (such as spontaneous subarachnoid hemorrhage) have elevated troponin and decreased LVEF.^[23]^ If these changes are complicated by systolic dysfunction, they can result in vasospasm, hypotension, and pulmonary edema, thereby increasing the risk of cerebral ischemia.^[23–27]^ In such patients, elevated troponin has high sensitivity and specificity for predicting cardiac dysfunction. Compared with traditional troponin, Hs-cTnT has higher sensitivity for predicting cardiac dysfunction, and therefore, it is significant for the screening and risk stratification of cardiovascular diseases.^[26]^ We revealed that the level of Hs-cTnT in patients with systolic dysfunction following Ims-TBI was higher than that in patients with no systolic dysfunction. Since the Hs-cTnT test was performed in the emergency department within 6 hours of admission (usually earlier than the time of echocardiography that was performed within 24 hours), it appears that myocardial function damage can occur during the early stages following admission of patients with TBI, which later affects the systolic function of the heart.

We also observed that patients with cardiac dysfunction after Ims-TBI had increased duration of mechanical ventilation and ICU LOS and higher in-hospital mortality compared with the no systolic dysfunction group. Further multivariate analysis showed that the development of systolic dysfunction was independently associated with in-hospital mortality in patients with Ims-TBI. These results indicate that the development of systolic dysfunction after Ims-TBI has an impact on the prognosis of these patients. This may be explained by the critical impact of cardiac output on cerebral blood flow.^[28]^ In patients with Ims-TBI and cardiac systolic dysfunction, events such as decreased cardiac output, hypotension, and arrhythmia during the hospitalization period may occur, which ultimately result in decreased cerebral blood flow.^[29–31]^ Decreased cerebral blood flow exacerbates secondary brain damage, thereby affecting the prognosis of the patient.^[15]^ It is precisely because the development of systolic dysfunction is related to the outcome of patients with Ims-TBI during the hospitalization that our study sought to identify risk factors that affect the occurrence of systolic dysfunction after TBI. We found that GCS, heart rate, and serum Hs-cTnT level on admission are independent risk factors that affect the development of cardiac dysfunction in patients with TBI. Further, we performed receiver operating characteristic curve analysis to determine the predictive value of various prognostic indicators in patients with Ims-TBI and systolic dysfunction. The results showed that low GCS and elevated serum Hs-cTnT level can predict systolic dysfunction in Ims-TBI patients. Our findings indicate that special attention should be paid to those Ims-TBI patients with a GCS of <7, high-sensitivity troponin >13.3 pg/mL, and a heart rate of >92 beats/min. They were more likely to develop cardiac systolic dysfunction. Echocardiography should be performed as soon as possible to accurately assess cardiac function in these patients.

Our study had several limitations. First, we did not evaluate the diastolic function of patients with Ims-TBI. The evaluation of the diastolic function of the heart is helpful in guiding the efficient fluid resuscitation and vasoactive drug therapy and in reducing the risk of pulmonary edema. Second, we did not dynamically evaluate the systolic function, which may be altered in patients with TBI. In addition, further studies are needed to clarify the time course of excessive catecholamine release in patients with TBI and how this is related to cardiac dysfunction. Finally, the relationship between these changes and the functions of other organ systems should be assessed.

## 5. Conclusions

Systolic dysfunction is a common complication in patients with Ims-TBI in the early postinjury period, even in those with no history of heart disease. Heart rate, GCS, and serum Hs-cTnT level on admission were independently associated with the development of systolic dysfunction following Ims-TBI. The combination of heart rate, GCS, and serum Hs-cTnT level on admission can better predict the occurrence of systolic dysfunction. The development of systolic dysfunction after Ims-TBI was independently associated with in-hospital outcomes. Therefore, it is necessary to pay attention to the severity of the head injury, relevant hemodynamic parameters, and myocardial damage biomarkers during the early stages of admission and to perform echocardiography when necessary to accurately assess cardiac function in patients with Ims-TBI. These practices will allow for early identification of possible TBI complications and, therefore, promote early intervention and improved outcomes in such patients.

## Acknowledgments

We thank all the participants for their contributions, institutional support and peer reviewers for their helpful comments on this paper.

## Author contributions

**Data curation:** Jungen Li, Yuzhu Miao, Xiaowen Xu, Bingyuan Zhou

**Formal analysis:** Jungen Li, Guoxing Zhang

**Methodology:** Jungen Li, Xiaowen Xu, Tingbo Jiang, Shiqi Lu

**Writing – original draft:** Jungen Li

**Writing – review & editing:** Jungen Li, Tingbo Jiang, Shiqi Lu

**Investigation:** Yuzhu Miao, Guoxing Zhang, Yanxia Guo

**Project administration:** Guoxing Zhang, Yanxia Guo, Tingbo Jiang, Shiqi Lu

**Resources:** Yanxia Guo, Bingyuan Zhou

**Software:** Yanxia Guo

**Validation:** Shiqi Lu

## References

[1] Maas AIR Menon DK Adelson PD.; InTBIR Participants and Investigators. Traumatic brain injury: integrated approaches to improve prevention, clinical care, and research. Lancet Neurol. 2017;16:987–1048. 29122524 10.1016/S1474-4422(17)30371-X

[2] Jiang JY Gao GY Feng JF. Traumatic brain injury in China. Lancet Neurol. 2019;18:286–95. 30784557 10.1016/S1474-4422(18)30469-1

[3] Stocchetti N Maas AI. Traumatic intracranial hypertension. N Engl J Med. 2014;370:2121–30. 24869722 10.1056/NEJMra1208708

[4] Meng L Hou W Chui J Han R Gelb AW. Cardiac output and cerebral blood flow: the integrated regulation of brain perfusion in adult humans. Anesthesiology. 2015;123:1198–208. 26402848 10.1097/ALN.0000000000000872

[5] Salem R Vallée F Dépret F. Subarachnoid hemorrhage induces an early and reversible cardiac injury associated with catecholamine release: one-week follow-up study. Crit Care. 2014;18:558. 25358417 10.1186/s13054-014-0558-1 PMC4245729

[6] Krishnamoorthy V Rowhani-Rahbar A Gibbons EF. Early systolic dysfunction following traumatic brain injury: a cohort study. Crit Care Med. 2017;45:1028–36. 28398926 10.1097/CCM.0000000000002404 PMC5433903

[7] Lang RM Badano LP Mor-Avi V. Recommendations for cardiac chamber quantification by echocardiography in adults: an update from the American Society of Echocardiography and the European Association of Cardiovascular Imaging. Eur Heart J Cardiovasc Imaging. 2015;16:233–70. 25712077 10.1093/ehjci/jev014

[8] Prathep S Sharma D Hallman M. Preliminary report on cardiac dysfunction after isolated traumatic brain injury. Crit Care Med. 2014;42:142–7. 23963125 10.1097/CCM.0b013e318298a890 PMC3841242

[9] Carney N Totten AM O’Reilly C. Guidelines for the management of severe traumatic brain injury, fourth edition. Neurosurgery. 2017;80:6–15. 27654000 10.1227/NEU.0000000000001432

[10] Mascia L Sakr Y Pasero D Payen D Reinhart K Vincent JL; Sepsis Occurrence in Acutely Ill Patients (SOAP) Investigators. Extracranial complications in patients with acute brain injury: a post-hoc analysis of the SOAP study. Intensive Care Med. 2008;34:720–7. 18175107 10.1007/s00134-007-0974-7

[11] Boland TA Lee VH Bleck TP. Stress-induced cardiomyopathy. Crit Care Med. 2015;43:686–93. 25565459 10.1097/CCM.0000000000000851

[12] Banki N Kopelnik A Tung P. Prospective analysis of prevalence, distribution, and rate of recovery of left ventricular systolic dysfunction in patients with subarachnoid hemorrhage. J Neurosurg. 2006;105:15–20. 16871878 10.3171/jns.2006.105.1.15

[13] Tahsili-Fahadan P Geocadin RG. Heart-brain axis: effects of neurologic injury on cardiovascular function. Circ Res. 2017;120:559–72. 28154104 10.1161/CIRCRESAHA.116.308446

[14] Rachfalska N Putowski Z Krzych LJ. Distant organ damage in acute brain injury. Brain Sci. 2020;10:1019. 33371363 10.3390/brainsci10121019 PMC7767338

[15] Krishnamoorthy V Mackensen GB Gibbons EF Vavilala MS. Cardiac dysfunction after neurologic injury: what do we know and where are we going? Chest. 2016;149:1325–31. 26836901 10.1016/j.chest.2015.12.014 PMC4944787

[16] Wallner M Duran JM Mohsin S. Acute catecholamine exposure causes reversible myocyte injury without cardiac regeneration. Circ Res. 2016;119:865–79. 27461939 10.1161/CIRCRESAHA.116.308687 PMC5026617

[17] Samuels MA. The brain-heart connection. Circulation. 2007;116:77–84. 17606855 10.1161/CIRCULATIONAHA.106.678995

[18] Clifton GL Ziegler MG Grossman RG. Circulating catecholamines and sympathetic activity after head injury. Neurosurgery. 1981;8:10–4. 7207763 10.1227/00006123-198101000-00003

[19] Krishnamoorthy V Rowhani-Rahbar A Chaikittisilpa N. Association of early hemodynamic profile and the development of systolic dysfunction following traumatic brain injury. Neurocrit Care. 2017;26:379–87. 28000133 10.1007/s12028-016-0335-x PMC5444944

[20] Krishnamoorthy V Chaikittisilpa N Kiatchai T Vavilala M. Hypertension after severe traumatic brain injury: friend or foe? J Neurosurg Anesthesiol. 2017;29:382–7. 27648804 10.1097/ANA.0000000000000370 PMC5357208

[21] Alali AS McCredie VA Golan E Shah PS Nathens AB. Beta blockers for acute traumatic brain injury: a systematic review and meta-analysis. Neurocrit Care. 2014;20:514–23. 24062229 10.1007/s12028-013-9903-5

[22] Silvani A Calandra-Buonaura G Dampney RA Cortelli P. Brain-heart interactions: physiology and clinical implications. Philos Trans A Math Phys Eng Sci. 2016;374:20150181. 27044998 10.1098/rsta.2015.0181

[23] Hravnak M Frangiskakis JM Crago EA. Elevated cardiac troponin I and relationship to persistence of electrocardiographic and echocardiographic abnormalities after aneurysmal subarachnoid hemorrhage. Stroke. 2009;40:3478–84. 19713541 10.1161/STROKEAHA.109.556753 PMC3680357

[24] Cai SS Bonds BW Hu PF Stein DM. The role of cardiac troponin I in prognostication of patients with isolated severe traumatic brain injury. J Trauma Acute Care Surg. 2016;80:477–83. 26910044 10.1097/TA.0000000000000916 PMC4770821

[25] De’Ath HD Rourke C Davenport R. Clinical and biomarker profile of trauma-induced secondary cardiac injury. Br J Surg. 2012;99:789–97. 22437496 10.1002/bjs.8728

[26] Jia X Sun W Hoogeveen RC. High-sensitivity troponin I and incident coronary events, stroke, heart failure hospitalization, and mortality in the ARIC study. Circulation. 2019;139:2642–53. 31030544 10.1161/CIRCULATIONAHA.118.038772 PMC6546524

[27] Temes RE Tessitore E Schmidt JM. Left ventricular dysfunction and cerebral infarction from vasospasm after subarachnoid hemorrhage. Neurocrit Care. 2010;13:359–65. 20945116 10.1007/s12028-010-9447-x

[28] Lie SL Hisdal J Høiseth L. Cerebral blood flow velocity during simultaneous changes in mean arterial pressure and cardiac output in healthy volunteers. Eur J Appl Physiol. 2021;121:2207–17. 33890157 10.1007/s00421-021-04693-6 PMC8260418

[29] Lim HB Smith M. Systemic complications after head injury: a clinical review. Anaesthesia. 2007;62:474–82. 17448059 10.1111/j.1365-2044.2007.04998.x

[30] Zou R Shi W Tao J. Neurocardiology: cardiovascular changes and specific brain region infarcts. Biomed Res Int. 2017;2017:5646348. 28758117 10.1155/2017/5646348 PMC5512017

[31] van der Wall EE van Gilst WH. Neurocardiology: close interaction between heart and brain. Neth Heart J. 2013;21:51–2. 23239452 10.1007/s12471-012-0369-4 PMC3547430

